# 培美曲塞在晚期非小细胞肺癌二线及以上治疗中的疗效分析

**DOI:** 10.3779/j.issn.1009-3419.2012.03.08

**Published:** 2012-03-20

**Authors:** 峻岭 李, 静 王, 学志 郝, 湘茹 张, 远凯 石

**Affiliations:** 100021 北京，中国医学科学院中国协和医科大学肿瘤医院内科 Cancer Institute and Hospital, Chinese Academy of Medical Sciences and Peking Union Medical College, Beijing 100021, China

**Keywords:** 肺肿瘤, 培美曲塞, 化疗, Lung neoplasms, Pemetrexed, Chemotherapy

## Abstract

**背景与目的:**

培美曲塞是晚期非小细胞肺癌（non-small cell lung cancer, NSCLC）推荐的标准二线治疗方案之一。目前二线治疗之后如何选择化疗药物尚无标准。本研究旨在观察培美曲塞治疗既往接受过多程治疗的晚期NSCLC的疗效和安全性。

**方法:**

回顾性分析2005年2月-2009年9月在中国协和医科大学肿瘤医院内科肺癌中心接受培美曲塞单药治疗，且既往接受过1个及以上方案治疗的37例晚期NSCLC的病例资料。

**结果:**

37例患者中二线治疗者13例（35.1%），三线及以上治疗者24例（64.9%）。疾病控制率为54.1%，其中1例（2.7%）完全缓解（双肺转移瘤消失），2例（5.4%）部分缓解，17例（45.9%）稳定，12例（32.4%）进展。中位无进展生存期为8.05个月，中位生存时间为19.29个月。毒副反应轻微。

**结论:**

培美曲塞用于晚期NSCLC二线及以上治疗耐受性较好，并且有生存获益，可推荐作为二线及以上晚期NSCLC患者治疗的选择。

## Abstract (en)

**Background and objective:**

Pemetrexed is a standard second-line therapy in advanced non-small cell lung cancer (NSCLC). Currently, cytotoxic treatments beyond second-line therapy are not available. This study evaluated the efficacy and safety of pemetrexed as a salvage regimen in heavily pretreated patients with NSCLC.

**Methods:**

Clinical data on 37 patients with advanced NSCLC who received pemetrexed as second-line therapy or beyond in Cancer Hospital of Peking Union Medical College from Feb 2005 to Sep 2009 were reviewed and analyzed retrospectively.

**Results:**

Thirteen patients (35.1%) received pemetrexed as second-line treatment, whereas 24 (64.9%) received it as third-line treatment or beyond. Complete response, partial response, stable disease and progressive disease were noted in 1 (2.7%), 2 (5.4%), 17 (45.9%) and 12 (32.4%) of the patients, respectively, with the disease control rate being 54.1%. The median duration of progression free survival was 8.05 months, whereas that of overall survival was 19.29 months.

**Conclusion:**

Pemetrexed is efficacious and tolerable as second-line therapy or beyond for advanced NSCLC and should thus be recommended for patients with this disease.

目前肺癌是世界范围内最常见的恶性肿瘤，其中非小细胞肺癌（non-small cell lung cancer, NSCLC）占80%以上，并且50%以上的患者在确诊时已是晚期。目前已有证据^[1, 2]^证实晚期NSCLC一线含铂方案化疗比最佳支持治疗能改善生存。但所有患者一线治疗后最终会出现进展，需要接受进一步的解救治疗。NCCN指南中推荐的二线治疗药物有多西他赛、培美曲塞、厄洛替尼、吉非替尼^[3-5]^。对于二线治疗之后的选择，有证据支持的仅有厄洛替尼^[5]^。在实际临床工作中二线治疗之后许多患者仍可选择化疗。培美曲塞是一种新型多靶点抗叶酸化疗药物，对包括NSCLC在内的多种肿瘤具有活性，主要作用机制为阻断DNA复制以及细胞分裂所需要的胸苷酸合成酶（thyidylate synthethase, TS）、甘氨酰胺核苷酸甲酰基转移酶（glycinamide ribonucleotide formyl transferase, GARFT）和二氢叶酸还原酶（ihydrofolate reductased, DHFR），使细胞分裂停止于S期，从而抑制肿瘤细胞的生长。由于培美曲塞具有良好的耐受性，二线治疗已证实疗效较好，因此亦被用于二线治疗之后的选择。本研究回顾性分析了中国医学科学院肿瘤医院肺癌中心2005年2月-2009年9月使用培美曲塞单药治疗，且既往接受过1个及以上方案治疗的晚期NSCLC患者资料，旨在了解其疗效及安全性。

## 1 材料和方法

### 1.1 病例选择

2005年2月-2009年9月在中国医学科学院肿瘤医院肺癌中心治疗，经组织学或细胞学证实并有可测量病灶的Ⅳ期NSCLC；患者必须接受过1个及以上方案的治疗，包括化疗及靶向治疗。

### 1.2 治疗情况

培美曲塞500 mg/m^2^，静脉输注，每21天重复。治疗前1周开始补充叶酸400 μg/d或含叶酸400 μg的复合维生素片剂，持续服用至末次培美曲塞治疗后的3周。首次培美曲塞治疗前1周给予维生素B_12_肌肉注射1, 000 μg，之后每9周注射1次至治疗结束。培美曲塞治疗前1天、当天及治疗后第1天给予地塞米松口服4 mg bid。

### 1.3 疗效评价及数据分析

每2周期化疗后进行1次CT复查，参照RECIST标准进行疗效评价。无进展生存期（progression free survival, PFS）定义为自首次培美曲塞治疗开始至疾病进展时间，未出现进展者记录为末次随访时间或死亡时间。总生存期（overall survival, OS）定义为自首次培美曲塞治疗开始至死亡时间或末次随访时间。随访截止时间为2011年12月19日。毒副反应按NCI-CTC 3.0标准进行评价。采用统计软件SPSS 17.0进行数据处理。

## 2 结果

### 2.1 基线特征

见表 1。符合条件的患者共37例，中位年龄52岁（30岁-73岁），男性18例，女性19例，非吸烟者21例（56.8%）。ECOG评分为0分-2分。腺癌29例，鳞癌5例，大细胞癌2例，1例NSCLC患者病理类型未明确。培美曲塞为二线治疗者有13例，为三线治疗者4例，四线13例，其它的7例均为四线以上治疗。一线方案为含铂方案者有34例（92%），1例患者行吉非替尼（商品名：易瑞沙）治疗。一线化疗的疗效：有记录的35例患者中，部分缓解（partial response, PR）12例（34.3%），稳定（stable disease, SD）17例（48.6%），进展（progressive disease, PD）6例（17.1%）。自确诊至开始接受培美曲塞治疗的中位时间为17.84个月。

**1:** 37例晚期非小细胞肺癌患者的基线特征 Basic characteristics of thirty-seven patients with advanced non-small cell lung cancer (NSCLC)

Characteristics	*n*	Second-line (*n*=13)	Third-line and beyond (*n*=24)
Median age (year)	52	52	51.5
Gender			
Male	18	6	12
Female	19	7	12
ECOG performance status			
0-1	32	11	21
2-3	2	0	2
Unkown	3	2	1
Smoking history			
Never	21	7	14
Former and current	13	6	7
Unkown	3	0	3
Histology			
Adenocarcinoma	29	11	18
Large-cell carcinoma	2	1	1
Squamous cell carcinoma	5	1	4
Unkown	1	0	1
First-line therapy			
Taxol-platinum	15	5	10
Vinorelbine-platinum	9	4	5
Gemcitabine-platinum	10	3	7
Others	3	1	2
Sencond-line therapy			
Tyrosine kinase inhibitors	-	-	10
Docetaxel	-	-	4
Docetaxel-based	-	-	5
Others	-	-	5
Best response to first-line therapy			
Partial response	12	6	6
Stable disease	17	3	14
Progressive disease	6	3	3
Unkown	2	1	1

### 2.2 培美曲塞疗效

见表 2。培美曲塞化疗的中位周期数为3个（1个-8个），16例（43.2%）患者接受了≥4个周期化疗。32例可评价病例，疾病控制率为54.1%，其中1例（2.7%）完全缓解（complete response, CR），双肺转移瘤消失，2例（5.4%）PR，17例（45.9%）SD，12例（32.4%）PD。中位PFS为8.05个月，中位OS为19.29个月。在二线治疗的13例患者中，7例患者培美曲塞治疗后接受了人表皮生长因子受体酪氨酸激酶抑制剂（epidermal growth factor receptor tyrosine kinase inhibitor, EGFR-TKI）治疗（吉非替尼或厄洛替尼），中位化疗周期数为4个（1个-7个），中位PFS为11.63个月，中位OS为26.35个月，12例可评价疗效，疾病控制率为76.9%，其中PR 1例，SD 9例。三线及以上治疗的24例患者中，10例患者在培美曲塞治疗前接受了EGFR-TKI（吉非替尼或厄洛替尼）的治疗，中位化疗周期数为2个（1个-8个），中位PFS为3.22个月，中位OS为17.71个月，疾病控制率为41.7%，其中1例CR，1例PR，8例SD。

**2:** 培美曲塞疗效 Response to pemetrexed

Item	Sencond-line	Third-line and beyond	Total
Response			
Complete response	0	1 (4.2%)	1 (2.7%)
Partial response	1 (7.7%)	1 (4.2%)	2 (5.4%)
Stable disease	9 (69.2%)	8 (33.3%)	17 (45.9%)
Progressive disease	2 (15.4%)	10 (41.7%)	12 (32.4%)
Disease control	10 (76.9%)	10 (41.7%)	20 (54.1%)
Not evaluable	1 (7.7%)	4 (16.7%)	5 (13.5%)
Progression free survival (range) (month)	11.63 (6.44-16.82)	3.22 (0-7.16)	8.05 (2.04-14.06)
Overall survival (range) (month)	26.35 (14.52-38.18)	17.71 (10.18-25.24)	19.29 (15.67-22.90)

### 2.3 毒副反应

见表 3。培美曲塞单药治疗的耐受性较好，本研究中3度及以上的不良反应主要为白细胞减少2例，中性粒细胞减少2例，血红蛋白减低1例，血小板减少1例。皮疹及胃肠道反应均在2度以下。发生3度不良反应的患者均为四线及以上治疗者。

**3:** 培美曲塞治疗的毒性反应 Toxicity profiles of pemetrexed treatment

Toxicity profiles	Sencond-line				Third-line and beyond		
	Grade 1/2	Grade 3	Grade 4		Grade 1/2	Grade 3	Grade 4
Leukopenia	6	0	0		5	2	0
Neutropenia	5	0	0		5	1	1
Anemia	5	0	0		7	1	0
Thrombocytopenia	0	0	0		2	0	1
Rash	1	0	0		0	0	0
Nausea/vomiting	4	0	0		15	0	0

## 3 讨论

影像学技术的发展能较早地发现转移性肺癌，例如无症状的颅内转移、骨转移及肾上腺转移等。这些患者在确诊时一般情况较好，可以接受多程治疗包括化疗及靶向治疗。培美曲塞是晚期NSCLC的标准二线治疗，也是非鳞癌标准的一线治疗，但在二线以上的患者中也经常使用。本项研究分析了培美曲塞用于二线及以上治疗的疗效及安全性。疾病控制率为54.1%，中位PFS为8.05个月，中位OS为19.29个月。与既往培美曲塞用于二线治疗的研究^[4]^相比，PFS相当而OS延长。单独分析二线治疗的患者，中位PFS及OS均延长。考虑造成这一结果的原因与本研究中非鳞癌NSCLC患者占大多数有关（86.5%），而这部分人群是培美曲塞治疗的优势人群^[6]^。此外该研究中的大多数患者在培美曲塞治疗后继续接受了后续治疗，54%的患者接受了EGFR-TKI治疗，并且本研究的入组例数偏少，可能造成二线治疗人群的PFS明显延长。进一步对比二线治疗及三线及以上治疗的人群，三线以上治疗人群中PFS为3.22个月，中位OS亦达到17.71个月，并不劣于二线治疗的疗效，与既往研究的结果相当^[7, 8]^。在BR.21研究^[5]^中厄洛替尼用作三线及以上治疗的患者占49.4%，厄洛替尼组中位OS为6.7个月，因此，本研究结果提示培美曲塞二线及以上治疗NSCLC的疗效不劣于厄洛替尼。

安全性方面培美曲塞单药治疗的耐受性较好，本研究中无3度非血液学毒性的发生，3度以上血液学毒性亦很低（5.4%），并且均发生在四线以上治疗的患者中，考虑与患者多程治疗后自身骨髓功能下降有关。

本项研究显示，晚期NSCLC患者二线及以后治疗选择培美曲塞可获益，并且耐受性较好。但目前的研究均为小样本回顾性分析，尚需进一步培美曲塞用于NSCLC三线及以上治疗的前瞻性研究来证实。
